# Gas Chromatography–Mass Spectrometry Quantification of 1,1-Dimethylhydrazine Transformation Products in Aqueous Solutions: Accelerated Water Sample Preparation

**DOI:** 10.3390/molecules26195743

**Published:** 2021-09-22

**Authors:** Mark S. Popov, Nikolay V. Ul’yanovskii, Dmitry S. Kosyakov

**Affiliations:** 1 Laboratory of Environmental Analytical Chemistry, Core Facility Center ‘Arktika’, Northern (Arctic) Federal University, 163002 Arkhangelsk, Russia; m.popov@narfu.ru (M.S.P.); d.kosyakov@narfu.ru (D.S.K.); 2 Federal Center for Integrated Arctic Research, 163000 Arkhangelsk, Russia

**Keywords:** 1,1-dimethylhydrazine, rocket fuel, transformation products, nitrogen-containing compounds, sample preparation, extraction, aqueous samples, accelerated water sample preparation, GC-MS

## Abstract

The use of highly toxic rocket fuel based on 1,1-dimethylhydrazine (UDMH) in many types of carrier rockets poses a threat to environment and human health associated with an ingress of UDMH into wastewater and natural reservoirs and its transformation with the formation of numerous toxic nitrogen-containing products. Their GC-MS quantification in aqueous samples requires matrix change and is challenging due to high polarity of analytes. To overcome this problem, accelerated water sample preparation (AWASP) based on the complete removal of water with anhydrous sodium sulfate and transferring analytes into dichloromethane was used. Twenty-nine UDMH transformation products including both the acyclic and heterocyclic compounds of various classes were chosen as target analytes. AWASP ensured attaining near quantitative extraction of 23 compounds with sample preparation procedure duration of no more than 5 min. Combination of AWASP with gas chromatography–mass spectrometry and using pyridine-*d*_5_ as an internal standard allowed for developing the rapid, simple, and low-cost method for simultaneous quantification of UDMH transformation products with detection limits of 1–5 μg L^−1^ and linear concentration range covering 4 orders of magnitude. The method has been validated and successfully tested in the analysis of aqueous solutions of rocket fuel subjected to oxidation with atmospheric oxygen, as well as pyrolytic gasification in supercritical water modelling wastewater from carrier rockets launch sites.

## 1. Introduction

The use of highly toxic rocket fuel based on 1,1-dimethylhydrazine (unsymmetrical dimethylhydrazine, UDMH) in many types of Russian, Indian, EU, and Chinese launch vehicles inevitably gives rise to a risk of environmental pollution. It can be associated with an ingress of rocket fuel into soils and water bodies in fall sites of spent rocket stages, emergency situations, as well as the disposal of UDMH containing wastewater generated at launch sites during storage, transportation of fuel, and refueling the carrier rockets [1,2]. The situation is complicated by the high reactivity of UDMH causing the rapid formation of a large number (up to several hundreds) of toxic nitrogen-containing products of its oxidative transformations upon contact with atmospheric air or reagents used in the detoxification of waters and soils [3,4,5]. In addition to well-known compounds such as N-nitrosodimethylamine (NDMA), 1,1,4,4-tetramethyltetrazene (TMT), N,N-dimethylformamide (DMF), 1-formyl-2,2-dimethylhydrazine (FADMH), and N,N-dimethylaminoacetonitrile (DMAAN), these include numerous heterocyclic compounds which are the final and most stable UDMH transformation products [4]. The identification and simultaneous quantification of a wide range of nitrogen-containing compounds of various classes is a challenging and still not fully resolved analytical task. HPLC-MS methods were successfully used for the determination of hydrazines and some products of their oxidation, for example, NDMA [6,7,8]. Given the high polarity of many nitrogen-containing compounds, their separation is impossible in reverse-phase HPLC mode, thus hydrophilic interaction liquid chromatography [9], stationary phases based on porous graphitized carbon [10], and analyte derivatization methods [11,12,13] were used to achieve acceptable chromatographic retention. Gas chromatography is a more universal solution for separating the widest possible range of UDMH transformation products (with the exception of hydrazines and some thermolabile compounds). In combination with mass spectrometric detection, it has found application for the search, identification, and determination of dozens of UDMH transformation products in soils [5,14,15,16,17,18,19].

The use of GC-MS for the analysis of aqueous samples is limited by the need to change the matrix, since the introduction of even small amounts of water into the chromatographic column leads to distortion of the shape of the chromatographic peaks, loss of separation efficiency, and instability of the analyte retention times. To solve this problem, liquid–liquid extraction (LLE) from a saturated saline solution [14,16,19], as well as solid-phase microextraction (SPME) from the vapor phase [20,21] were used. Despite the possibility of significant preconcentration of analytes, these methods also have a number of disadvantages. These include the relative complexity and duration of the sample preparation procedure, the difficulty of extracting the most polar analytes from aqueous solutions, as well as the competition of analytes and matrix components for sorption centers of SPME fiber and, as a consequence, significant matrix effects requiring complicated matrix-matching calibration procedures and isotopically labelled internal standards. To overcome them, approaches based on the complete removal of free water from the sample with the displacement of analytes into an organic solvent seem promising. To absorb water, zeolites [22], as well as salts easily forming crystalline hydrates (for example, sodium sulfate) can be used. The latter approach was developed by Polyakova et al. [23] and named AWASP (accelerated water sample preparation). It was successfully used for the determination of polyaromatic hydrocarbons [23], and, subsequently, for a variety of volatile and semi-volatile organic compounds, including a number of nitrogen-containing analytes [24,25,26]. AWASP has proven to be a simple and inexpensive alternative to purge-and-trap and SPME techniques for tasks that do not require high analyte preconcentration degree.

The aim of this study is to expand the boundaries of AWASP application for polar nitrogen-containing compounds and to develop on this basis the simple and rapid method for the determination of a wide range of UDMH transformation products in aqueous samples by GC-MS.

## 2. Results and Discussion

### 2.1. Extraction Efficiency and Optimization

**Figure 1:**
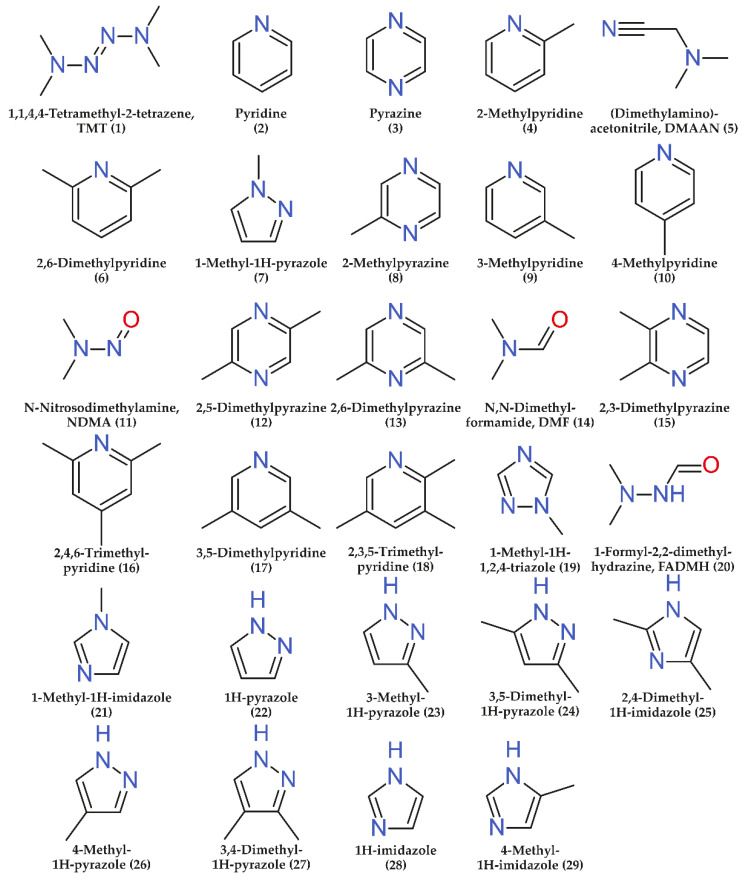
Structural formulas of the target analytes (numbers in parenthesis correspond to the serial numbers of analytes in Figure 2 and tables).

Twenty-nine commercially available nitrogen-containing compounds (Figure 1) which are known UDMH transformation products [4,5,27] were chosen as analytes. Among them, there are both the acyclic components mentioned in the Introduction (NDMA, TMT, DMF, FADMH, and DMAAN) and a number of heterocyclic compounds (1-methyl-1H-1,2,4-triazole, 1H-pyrazole, pyrazine, pyridine, 1H-imidazole, and their methyl derivatives). A complete list of analytes, as well as some of their physico-chemical properties are presented in Supplementary Materials (Table S1).

**Figure 2:**
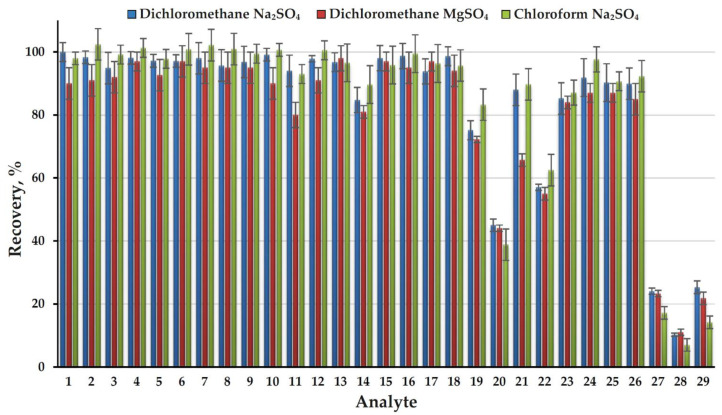
Recoveries of analytes (extraction efficiency) obtained with three extractant-dehydrating agent systems.

To determine the recoveries of analytes (extraction efficiency) an approach based on comparing the areas of chromatographic peaks of each component when analyzing an extract from an aqueous standard sample with a concentration of 10 mg L^−1^ and a standard solution in an extractant with the same concentration was used. The obtained results (Figure 2) demonstrate the high efficiency of AWASP when using dichloromethane as an extractant, a close to quantitative extraction was achieved for 23 of 29 analytes (recovery > 80%). Only four analytes (FADMH, 4-methyl-1H-imidazole, 3,4-dimethyl-1H-pyrazole, and 1H-imidazole) have recoveries of less than 50% and the minimum value (10%) was observed for the latter compound. In addition to the high dipole moment, a feature of such analytes is the presence of a secondary amine group in their structure. Possessing an N-H group is also characteristic of the fifth compound with a significantly reduced recovery (57%): pyrazole. For these compounds the observed recoveries are poorly correlated with the Log*P* values (Table S1 in Supplementary Materials). This is due to the fact that the efficiency of extraction into an organic solvent in the absence of free water is determined not by the partition mechanism, but the capability of analytes for binding to salt ions in solid phase and water molecules in crystalline hydrates due to specific donor-acceptor interactions. It should be noted that the use of AWASP made it possible to efficiently extract from an aqueous solution such most important highly polar (Log*P* < 0) UDMH transformation products as pyrazine, NDMA, 1-methyl-1H-1,2,4-triazole, and even DMF (Log*P* = –1.01, recovery 85%), for which common liquid–liquid extraction with dichloromethane under normal conditions does not provide acceptable results.

Due to the exceptional simplicity of the extraction procedure and complete removal of the aqueous matrix, the possibilities for optimizing AWASP conditions are rather limited. While in liquid–liquid extraction of nitrogen-containing compounds the decisive role belongs to their conversion into the molecular form by alkalization of the solution, in the case of AWASP no noticeable differences in the recoveries were observed in the wide pH range (5–12) of aqueous phase (Supplementary, Figure S1). This effect is quite expected due to the absence of an aqueous phase at the end of the extraction procedure and thus inability of analytes to exist in the cationic form. The most important parameters for optimizing the AWASP procedure are the nature and volume of the organic solvent and the water-binding reagent. The replacement of dichloromethane with chloroform did not lead to a significant change in the extraction efficiency for the overwhelming majority of analytes. At the same time, for the four poorly extractable compounds mentioned above, it caused a substantial decrease in the extraction efficiency (Figure 2). Obviously, an increase in the volume of the extractant leads to an increase in the recoveries of poorly extractable analytes; however, in order to maintain an acceptable sensitivity of the analysis, an additional stage of extract evaporation is required. This complicates the sample preparation procedure, making the use of AWASP meaningless. Reducing the volume of extractant to less than 500 μL causes difficulties in separating the extract from the solid phase and lower reproducibility of the analysis. The use of magnesium sulfate as a dehydrating agent instead of sodium sulfate in most cases also results in a slight decrease in the extraction efficiency (Figure 2). Other available drying agents (CaO, BaO, Mg(ClO_4_)_2_, molecular sieves) are characterized by a lower capacity, significant exothermic effects of interaction with water, and, in the case of zeolites, the capability for unwanted adsorption of some polar analytes. Based on the results of preliminary testing, they were rejected for use in further research. Thus, to achieve maximum efficiency and simplicity of sample preparation when analyzing 500-μL aqueous samples, the dichloromethane (500 μL)–sodium sulfate system can be recommended. It was used in this study for further development of the analytical method.

### 2.2. Analytical Method and Its Validation

The combination of AWASP with the analysis of the extracts by GC-MS made it possible to develop a simple and reliable method for the determination of UDMH transformation products in water bodies. The key issue in this case was to ensure the reproducibility of the analysis, which is negatively affected by the incomplete separating the extractant liquid phase from the solid salt, as well as by the difficulty of mass transfer between the two phases. The problem was successfully overcome by using an internal isotope-labelled standard introduced into an aqueous sample before extraction. As such a compound, the deuterated pyridine (pyridine-*d*_5_) was used due to its availability as a high-purity preparation (a widely used solvent for NMR spectroscopy), stability, low cost, and quantitative extraction from an aqueous solution during AWASP.

Limits of detection (LODs) and quantification (LOQs) were determined using the signal-to-noise ratio criteria 3:1 and 10:1, respectively, and confirmed in the analyses of model solutions of analytes with concentrations close to LOQ. For most analytes, the achieved instrumental LODs lie in a rather narrow range of 1–5 μg L^−1^ (Table 1) only slightly differing for the single ion monitoring (SIM) and multiple reaction monitoring (MRM) mass spectrometric detection modes. An exception is DMAAN, for which the SIM mode provides a loss in sensitivity by almost an order of magnitude due to the increased background signal and noise level at *m/z* 84. Naturally, when analyzing real objects with a complex matrix, the use of the MRM mode is preferable for all analytes due to possible interferences from coeluting isobaric or isomeric matrix components in the SIM mode, which results in increased LOD values. It is natural that poorly extractable analytes (FADMH, 4-methyl-1H-imidazole, 3,4-dimethyl-1H-pyrazole, and 1H-imidazole) are characterized by higher LODs (10–40 μg L^−1^). This prevents AWASP from being recommended for reliable quantification of these compounds at low levels.

In both SIM and MRM modes the obtained calibration curves were linear (*r*^2^ = 0.999) in the analyte concentration range covering at least four orders of magnitude, the upper limit of which exceeds 50 mg L^−1^. The exception is poorly extractable compounds and, especially, 4-methyl-1H-imidazole (*r*^2^ = 0.973 in SIM mode), which are distinguished by a larger point scattering about the calibration curve.

Due to the use of internal standard, the developed method demonstrates a high reproducibility. The intra-day and inter-day precision estimated by calculating the relative standard deviation (RSD) for the analysis of standard sample with concentration close to LOQ in six replicates lies in the ranges of 4–10% and 6–15%, respectively (Table 2). As can be seen, the values close to the upper limits of these ranges were observed for poorly extractable analytes.

The accuracy of the developed method was estimated by spike recovery test using real samples of river water and peat bog soil extract (Samples 1 and 2) at three concentration levels—20, 2, and 0.2 mg L^−1^ (n = 3, P = 0.95). The use of lower concentrations, close to LOQ, is not justified due to the high reactivity of many nitrogen-containing compounds and the occurrence of reactions of their binding with dissolved organic matter in natural waters, which leads to inadequate characteristics of the method accuracy [10,15]. The obtained results (Supplementary, Tables S2–S4) clearly confirm the substantial effect of natural organic matter on the recoveries of analytes. While in deionized water the accuracy is 100 ± 5% regardless of concentration; it significantly decreases in the river water matrix when going to a level of 0.2 mg L^−1^, while remaining in acceptable limits. The analysis of the peaty soil extract shows the low accuracy (spike recovery < 80%) for half of the analytes at the level of 0.2 mg L^−1^. Moreover, at the spiked concentrations close to LOQ some of the analytes could not be detected, while for the rest the results of analysis turned out to be irreproducible. Therefore, the method should be used with caution when analyzing aqueous samples with extremely high natural organic matter content. Nevertheless, this problem does not limit the application of the method for the determination of free forms of nitrogen-containing analytes, which is the most urgent problem for environmental applications.

The high robustness of the method is based on the exceptional simplicity of extraction procedure, complete removal of water interfering GC-MS analysis, and using the internal standard. It was confirmed by the successful analyses of several hundreds of real samples performed in our laboratory without deterioration of chromatographic separation of analytes and noticeable changing in the analytical characteristics of the method.

In general, the developed method differs from those previously described in the literature by exceptional simplicity and speed, as well as the minimum consumption of reagents and the possibility of implementing sample preparation directly in the field with subsequent delivery of extracts to the laboratory for GC-MS analysis. Due to not using the sample preconcentration during AWASP, the sensitivity of the method is inferior to the approaches based on the use of SPME; however, it provides the detection of even the most toxic of the studied compounds, NDMA, at the level of 0.5 MPC (the established level in Russia and Kazakhstan MPC in natural water is 10 μg L^−1^). Despite the much simpler extraction procedure, the LODs achieved are close to the values reported earlier by Ul’yanovskii et al. [14] and obtained using liquid–liquid extraction with acetonitrile from aqueous samples saturated with sodium chloride as salting-out agent.

### 2.3. Analyses of Real Samples

Aqueous solutions of 1,1-dimethylhydrazine transformation products obtained by oxidizing rocket fuel with air oxygen for a long time, as well as a result of its decomposition by pyrolysis in supercritical water [27,28,29], which has prospects for use in purification wastewater from launch sites, where chosen as real samples for testing the developed method. Eight and twenty-two compounds from the list of target analytes were found in the studied Samples 3 and 4, respectively (Table 3). Their identification was confirmed by the coincidence of the retention times and the ratios of the signal intensities of the quantifier and qualifier ions (ion transitions) for the real sample and the standard (Table 4).

In sample 3, the bulk (more than 99%) of the UDMH transformation products is accounted for by two compounds, NDMA (31 mg L^−1^) and 1-methyl-1H-1,2,4-triazole (49 mg L^−1^). The rest of the detected analytes were in trace concentrations ranging from LOQ to 100 μg L^−1^. An exception is DMF, the concentration of which was 218 mg L^−1^. It should be noted that Sample 3 contained large amounts of the initial UDMH and, apparently, the more complex transformation products [30], the formation of which is facilitated by mild oxidation conditions and a low degree of rocket fuel conversion.

A completely different picture is observed for UDMH pyrolysis products formed under extremely harsh conditions of supercritical water treatment. This sample exhibits a wide variety of the resulting products along with the complete absence of the parent UDMH [27]. Among them, in addition to NDMA and 1-methyl-1H-1,2,4-triazole, 1-Methyl-1H-Pyrazole, DMF, and FADMH predominate. The latter is the main product, which accounts for a half of the total content of the detected compounds. It is noteworthy that Sample 4 contains a large number of heterocyclic compounds, primarily pyridines, pyrazoles, and triazoles. Due to aromaticity, such structures have a lower free energy and thus the greatest stability. This makes nitrogen-containing heterocyclic compounds the end products of the transformation of UDMH, the formation of which is facilitated by harsh conditions.

The obtained results showed that the AWASP-GC-MS method can be successfully applied to study the chemical composition of UDMH containing wastewaters, as well as to control the technological processes of their treatment.

## 3. Materials and Methods

### 3.1. Analytes, Reagents and Materials

All analytes were purchased from Sigma-Aldrich (Sent-Louis, USA) and Alfa Aesar (Karlsruhe, Germany) and had a purity of ≥ 97%. The exceptions were NDMA and TMT, purchased from Ecoanalitika (Moscow, Russia) as certified standard solutions in acetonitrile with a concentration of 1 mg mL^−1^ as well as FADMH synthesized from UDMH and ethyl formate by the known procedure [31]. Deuterated pyridine (pyridine-*d*_5_, > 99.9%, Deutero GmbH, Kastellaun, Germany) was used as internal standard.

Dichloromethane (> 99.8%, PanReac, Barcelona, Spain) and HPLC grade chloroform (Komponent-Reaktiv, St. Petersburg, Russia) were used as extractants and for the preparation of analyte solutions. Methanol (HPLC gradient grade, Khimmed, Russia) was used to prepare working solutions. Aqueous solutions of standard samples, as well as model mixtures of analytes, were prepared using ultrapure Milli-Q water with a resistivity of 18.2 MΩ cm.

Anhydrous sodium and magnesium sulfates (99%, PanReac, Barcelona, Spain), 50% aqueous sodium hydroxide solution (extra pure, Komponent-Reaktiv, Moscow, Russia) were used in the extraction procedure.

The stock solutions of the analytes (except for NDMA and TMT) and the internal standard were prepared in methanol with a concentration of 10,000 mg L^−1^ from precisely weighed portions and stored at 4 °C for no more than a week. A working solution of analytes mixture with concentrations of each component of ~400 mg L^−1^ and pyridine-*d*_5_ with a concentration of 1 mg L^−1^ was prepared daily by mixing the stock solutions and diluting with water. Calibration solutions (10–50,000 μg L^−1^ for most analytes) were prepared by successive dilutions of the working solution with water. A model solution used to determine the extraction efficiency was prepared by mixing the stock solutions and sequential dilutions with dichloromethane (chloroform).

### 3.2. Real Objects

The following natural water samples knowingly not containing analytes were used to assess method accuracy and matrix interferences:

- *Sample 1*. River water (Northern Dvina River) with a salinity of 140 mg L^−1^ and a dissolved organic carbon content of 13 mg L^−1^.
- *Sample 2*. Water extract of peat bog soil, typical for landing places of launch vehicle’s spent stages in the European North of Russia. A soil sample weighing 1 g was placed in a 20 mL glass vial and poured with 10 mL of deionized water, then suspended with continuous vigorous stirring on a vortex (1500 rpm) for 20 min. After settling for two days and centrifugation the aqueous solution was separated and stored at 4 °C for no more than one week.

The following aqueous samples were used as real objects containing the analytes under study:

- *Sample 3*. An aqueous solution of 1,1-dimethylhydrazine with an initial concentration of 7000 mg L^−1^, which was in contact with air for 4 years and thus underwent significant oxidative conversion. The solution was yellow in color.
- *Sample 4*. An aqueous solution of 1,1-dimethylhydrazine with an initial concentration of 1000 mg L^−1^ subjected to pyrolytic gasification in supercritical water for 2 h at 600 °C [27]. This sample represents a product of detoxification of rocket fuel containing wastewater.

### 3.3. AWASP Procedure

A 500-μL portion of aqueous sample was placed in a 4-mL screw-cap glass vial, then 10 μL of an internal standard (pyridine-*d*_5_) solution with a concentration of 1 mg L^−1^ and 500 μL of dichloromethane (chloroform) were added using a micro syringe. Finely ground anhydrous sodium sulfate (or magnesium sulfate) of was added in small portions (the total amount of salt was ~1.5 g) with constant shaking on a vortex for ~3 min until complete binding of water is achieved. From the remaining liquid organic layer (350–400 μL) a 200-μL aliquot of was taken with micro syringe, transferred into a 1.5 mL vial with a 250-μL conical glass insert, and placed into an autosampler of the GC–MS system. The total duration of the sample preparation procedure was ~5 min. When determining the recovery of analytes (extraction efficiency), no internal standard was introduced.

### 3.4. GC-MS (MS/MS) Analyses

Shimadzu GCMS-TQ8040 gas chromatography–tandem mass spectrometry system (Kyoto, Japan) with triple quadrupole mass analyzer, split/splitless injector, and AOC-5000 Plus robotic autosampler was used in GC-MS and GC-MS/MS analyses. Chromatographic separation was achieved on an HP-INNOWax fused silica capillary column (Agilent Technologies, Santa Clara, USA), 30 m × 0.25 mm (i.d.), 0.25 μm film thickness, with a polyethylene glycol polar stationary phase providing good separation of nitrogen-containing compounds with minimal distortion of the chromatographic peaks. High-purity (99.9999%) helium (NIIKM, Moscow, Russia) with a flow rate of 1.2 mL min^−1^ was used as a carrier gas. Split (5:1) injection mode with a sample volume of 1 μL was used. Column thermostat was programmed from 40 °C (held for 3 min) to 240 °C (held for 5 min) at the 10 °C min^−1^ ramp. The total duration of the analysis was 28 min. The temperatures of the ion source and transfer line were 230 and 240 °C, respectively. Detection was performed using electron ionization (70 eV) in SIM and MRM modes. Argon (99.999%) was used as a collision gas in MRM mode. For each analyte, signals of two ions (or two MRM transitions) were recorded, one of which (with a more intense signal) was used for quantitative analysis, and the other as a qualifier ion (MRM transition) to increase the reliability of identifying analytes in real objects (Table 4). Collision energy (CE) for each MRM transition was optimized automatically in preliminary experiments to achieve maximum signal intensity. Two-step timed data acquisition program with simultaneous detection of 18 and 11 analytes in retention time (*t*_R_) ranges of 0–12 min and 11–23 min, respectively, was used. The signal accumulation time for each ion (MRM transition) was 30 ms. The control of the GC-MS system, collection and primary data processing were carried out using the LabSolutions software (Shimadzu, Kyoto, Japan). An example of the analytes model mixture chromatogram is presented in Supplementary (Figure S2).

**Table 4:** GC-MS parameters for SIM and MRM detection modes.

No	Analyte	t_R_, min	SIM		MRM			
			Quantifier ion, *m/z*	Qualifier ion *m/z*	Quantifier		Qualifier	
					*m/z*	CE, eV	*m/z*	CE, eV
1	TMT	5.91	116	72	116→72	6	72→44	3
2	Pyridine	7.00	79	52	79→52	15	52→50	9
3	Pyrazine	7.54	80	53	80→53	12	80→51	39
4	2-Methylpyridine	7.75	93	66	93→66	15	93→78	18
5	DMAAN	8.04	84	58	83→42	6	84→57	6
6	2,6-Dimethylpyridine	8.18	107	66	107→65	21	107→92	18
7	1-Methyl-1H-pyrazole	8.21	82	54	82→54	18	82→42	21
8	2-Methylpyrazine	8.50	94	67	94→67	12	67→40	6
9	3-Methylpyridine	9.00	93	66	93→66	15	93→39	36
10	4-Methylpyridine	9.38	93	66	93→66	15	93→39	36
11	NDMA	9.51	74	42	74→44	6	74→42	21
12	2,5-Dimethylpyrazine	9.48	108	42	108→42	18	108→81	9
13	2,6-Dimethylpyrazine	9.77	108	42	108→42	18	108→40	18
14	DMF	9.82	73	44	73→44	6	73→58	6
15	2,3-Dimethylpyrazine	9.77	108	67	108→67	12	67→40	6
16	2,4,6-Trimethylpyridine	10.10	121	79	121→79	15	79→77	12
17	3,5-Dimethylpyridine	10.78	107	79	107→92	15	106→77	15
18	2,3,5-Trimethylpyridine	11.70	121	106	121→77	33	120→77	18
19	1-Methyl-1H-1,2,4-triazole	13.18	83	56	83→56	6	83→55	18
20	FADMH	14.06	59	43	59→44	9	59→43	15
21	1-Methyl-1H-imidazole	14.44	82	54	82→55	12	82→42	18
22	1H-pyrazole	15.96	68	41	68→41	15	67→40	12
23	3-Methyl-1H-pyrazole	16.35	81	82	82→54	18	81→54	6
24	3,5-Dimethyl-1H-pyrazole	16.80	95	96	95→41	15	95→68	6
25	2,4-Dimethyl-1H-imidazole	17.49	96	95	95→41	18	95→68	6
26	4-Methyl-1H-pyrazole	16.93	82	81	82→55	12	81→54	6
27	3,4-Dimethyl-1H-pyrazole	19.87	95	96	96→54	21	95→68	6
28	1H-imidazole	20.30	68	55	68→41	15	67→40	6
29	4-Methyl-1H-imidazole	20.42	82	81	82→54	18	81→54	6

## 4. Conclusions

Accelerated water sample preparation (AWASP) technique with complete water removal ensures the achievement of high efficiency of extraction of rocket fuel 1,1-dimethylhydrazine polar nitrogen-containing transformation products from aqueous matrices. An exception is some secondary amines (FADMH, 4-methyl-1H-imidazole, 3,4-dimethyl-1H-pyrazole, and 1H-imidazole) with the reduced recoveries lying in the range of 10–50%. Due to the binding of water to crystalline hydrates, the extraction efficiency does not depend on the pH of the studied aqueous samples. The combination of AWASP with GC-MS (MS/MS) analysis made it possible to develop and validate a rapid method for the simultaneous determination of twenty-five 1,1-dimethylhydrazine transformation products of various classes with detection limits of 1-5 μg L^−1^ and linear concentration range covering 4 orders of magnitude, characterized by small volumes (0.5 mL) of aqueous sample and extractant (dichloromethane), exceptional simplicity and low cost of sample preparation. The developed method has been successfully tested in the analysis of aqueous solutions of rocket fuel subjected to oxidation with atmospheric oxygen, as well as pyrolytic gasification in supercritical water modelling wastewater from carrier rockets launch sites. When analyzing natural waters, it is necessary to take into account that extraction efficiency is significantly reduced in the presence of high concentrations of natural organic matter due to the binding of nitrogen-containing compounds. This phenomenon requires further studies for reliable differentiating free and bonded forms of analytes in natural objects.

## Figures and Tables

**Table 1:** The key specifications of the developed AWASP-GC-MS method and parameters of calibration curve (S_a_/S_is_ *= aC_a_*), linear range, limits of detection, and quantification (µg L^−1^) for SIM and MRM detection modes.

No	Analyte	SIM				MRM				Linear Range
		a	r^2^	LOD	LOQ	a	r^2^	LOD	LOQ	
1	TMT	0.477	0.999	3.1	10	0.215	0.999	4.7	16	LOQ–50
2	Pyridine	0.854	0.999	2.1	7.0	0.840	0.999	0.7	2.4	LOQ–58
3	Pyrazine	0.721	0.999	2.0	6.5	0.461	0.999	2.7	9.1	LOQ–58
4	2-methyl-Pyridine	0.963	0.999	2.1	7.1	0.373	0.999	3.2	11	LOQ–71
5	DMAAN	0.359	0.999	26	88	0.227	0.999	3.7	12	LOQ–54
6	2,6-Dimethylpyridine	1.16	0.999	1.0	3.2	0.273	0.999	2.4	8.1	LOQ–56
7	1-Methyl-1H-Pyrazole	0.865	0.999	2.3	7.7	0.198	0.999	2.5	8.2	LOQ–58
8	2-Methylpyrazine	0.898	0.999	2.1	6.9	0.503	0.999	1.9	6.4	LOQ–59
9	3-methylpyridine	0.905	0.999	3.2	11	0.344	0.999	4.7	15	LOQ–58
10	4-methylpyridine	0.980	0.999	3.1	10	0.427	0.999	4.0	13	LOQ–60
11	NDMA	0.507	0.999	4.6	15	0.307	0.999	4.8	16	LOQ–50
12	2,5-Dimethylpyrazine	0.910	0.999	2.0	6.8	0.274	0.999	2.0	6.5	LOQ–57
13	2,6-Dimethylpyrazine	1.21	0.999	1.5	5.1	0.285	0.999	1.9	6.4	LOQ–53
14	DMF	0.496	0.999	5.5	18	0.242	0.999	3.0	9.9	LOQ–57
15	2,3-Dimethylpyrazine	0.904	0.999	1.9	6.2	0.568	0.999	1.2	4.0	LOQ–60
16	2,4,6-Trimethylpyridine	1.38	0.999	1.0	3.2	0.352	0.999	1.3	4.4	LOQ–55
17	3,5-Dimethylpyridine	1.08	0.999	1.2	4.0	0.243	0.999	2.9	9.8	LOQ–57
18	2,3,5-Trimethylpyridine	1.13	0.999	1.2	3.8	0.160	0.999	2.0	6.7	LOQ–54
19	1-Methyl-1H-1,2,4-triazole	0.432	0.999	5.7	19	0.492	0.998	2.1	7.2	LOQ–63
20	FADMH	0.136	0.995	16	54	0.095	0.994	17	56	LOQ–59
21	1-Methyl-1H-imidazole	0.765	0.998	3.3	11	0.285	0.998	2.1	7.1	LOQ–63
22	1H-pyrazole	0.500	0.998	3.3	11	0.257	0.999	6.3	20	LOQ–57
23	3-Methyl-1H-pyrazole	0.411	0.999	5.0	17	0.127	0.999	2.8	9.4	LOQ–59
24	3,5-Dimethyl-1H-pyrazole	0.650	0.999	3.9	13	0.143	0.999	4.6	15	LOQ–58
25	2,4-Dimethyl-1H-imidazole	0.640	0.999	4.0	13	0.136	0.999	4.1	13	LOQ–58
26	4-Methyl-1H-pyrazole	0.490	0.999	4.1	14	0.146	0.999	4.4	15	LOQ–54
27	3,4-Dimethyl-1H-pyrazole	0.158	0.991	21	68	0.120	0.990	10	33	LOQ–56
28	1H-imidazole	0.225	0.991	21	71	0.302	0.992	37	120	LOQ–58
29	4-methyl-1H-Imidazole	0.300	0.973	11	36	0.153	0.980	26	87	LOQ–56

**Table 2:** Intra-day (24 h, *n* = 6) and inter-day (48 h, *n* = 6) precision of the developed AWASP-GC-MS method (MRM detection mode).

No	Analyte	RSD, %	
		Intra-Day	Inter-Day
1	TMT	5	6
2	Pyridine	5	8
3	Pyrazine	6	8
4	2-Methylpyridine	7	11
5	DMAAN	8	10
6	2,6-Dimethylpyridine	8	9
7	1-Methyl-1H-pyrazole	6	7
8	2-Methylpyrazine	8	10
9	3-Methylpyridine	5	11
10	4-Methylpyridine	5	10
11	NDMA	4	8
12	2,5-Dimethylpyrazine	4	8
13	2,6-Dimethylpyrazine	4	8
14	DMF	4	6
15	2,3-Dimethylpyrazine	5	11
16	2,4,6-Trimethylpyridine	6	13
17	3,5-Dimethylpyridine	5	10
18	2,3,5-Trimethylpyridine	7	9
19	1-Methyl-1H-1,2,4-triazole	8	11
20	FADMH	10	15
21	1-Methyl-1H-imidazole	8	12
22	1H-pyrazole	5	9
23	3-Methyl-1H-pyrazole	6	8
24	3,5-Dimethyl-H-pyrazole	4	9
25	2,4-Dimethyl-1H-imidazole	8	10
26	4-Methyl-1H-pyrazole	6	10
27	3,4-Dimethyl-1H-pyrazole	5	10
28	1H-imidazole	8	13
29	4-Methyl-1H-imidazole	10	15

**Table 3:** Results of AWASP-GC-MS (MRM detection mode) analysis of real samples.

No	Analyte	Concentration, µg L^−1^	
		Sample 3	Sample 4
1	TMT	n.d.*	19 ± 1
2	Pyridine	3.2 ± 0.8	403 ± 27
3	Pyrazine	5.4 ± 0.1	31 ± 2
4	2-Methylpyridine	n.d.	125 ± 14
5	DMAAN	n.d.	n.d.
6	2,6-Dimethylpyridine	n.d.	7.4 ± 1.9
7	1-Methyl-1H-pyrazole	95 ± 9	2180 ± 120
8	2-Methylpyrazine	5.9 ± 0.2	122 ± 5
9	3-Methylpyridine	n.d.	274 ± 21
10	4-Methylpyridine	n.d.	11 ± 1
11	NDMA	31,200 ± 1800	3590 ± 140
12	2,5-Dimethylpyrazine	n.d.	4.5 ± 0.2
13	2,6-Dimethylpyrazine	n.d.	15 ± 1
14	DMF	220 ± 50	5920 ± 200
15	2,3-Dimethylpyrazine	n.d.	8.0 ± 1.0
16	2,4,6-Trimethylpyridine	n.d.	3.4 ± 1.1
17	3,5-Dimethylpyridine	n.d.	9.7 ± 1.4
18	2,3,5-Trimethylpyridine	n.d.	5.2 ± 1.9
19	1-Methyl-1H-1,2,4-triazole	48,700 ± 7400	10,500 ± 220
20	FADMH	n.d.	25,100 ± 2100
21	1-Methyl-1H-imidazole	n.d.	430 ± 35
22	1H-pyrazole	25 ± 7	30 ± 3
23	3-Methyl-1H-pyrazole	n.d.	8.8 ± 3.7
24	3,5-Dimethyl-1H-pyrazole	n.d.	n.d.
25	2,4-Dimethyl-1H-imidazole	n.d.	n.d.
26	4-Methyl-1H-pyrazole	n.d.	n.d.
27	3,4-Dimethyl-1H-pyrazole	n.d.	n.d.
28	1H-imidazole	n.d.	n.d.
29	4-Methyl-1H-imidazole	n.d.	n.d.

* n.d.—not detected.

## Data Availability

The data presented in this study are available in the article and Supplementary Materials.

## Supplementary Materials

The following are available online, Figure S1: Comparison of the analyte recoveries in AWASP at aqueous phase pH 5 and 12 (extractant—dichloromethane, dehydrating agent—Na_2_SO_4_), Figure S2: GC-MS chromatogram (SIM detection mode) of the model mixture of target analytes (peak numbers correspond to compound numbers in tables), Table S1: List of analytes and their physico-chemical properties, Table S2: Accuracy of the developed AWASP-GC-MS method estimated by spike recovery test at 20 mg L^−1^ level with three aqueous matrices: Milli-Q water, river water (Sample 1), and peat bog soil aqueous extract (Sample 2), Table S3: Accuracy of the developed AWASP-GC-MS method estimated by spike recovery test at 2 mg L^−1^ level with three aqueous matrices: Milli-Q water, river water (Sample 1), and peat bog soil aqueous extract (Sample 2), Table S4: Accuracy of the developed AWASP-GC-MS method estimated by spike recovery test at 0.2 mg L^−1^ level with three aqueous matrices: Milli-Q water, river water (Sample 1), and peat bog soil aqueous extract (Sample 2).

## Sample Availability

Samples of the compounds are not available from the authors.
