# Supplementary material for: Gas Chromatography–Mass Spectrometry Quantification of 1,1-Dimethylhydrazine Transformation Products in Aqueous Solutions: Accelerated Water Sample Preparation
Source: Molecules. 2021 Sep 22;26(19):5743. doi: 10.3390/molecules26195743 (PMC8510043; doi:10.3390/molecules26195743)
Supplement: Supplementary file 1 [file molecules-26-05743-s001.zip › molecules-1371596-supplementary.pdf]

Supplementary Materials

Gas Chromatography–Mass Spectrometry Quantification of 1,1-Dimethylhydrazine Transformation Products in Aqueous Solutions: Accelerated Water Sample Preparation

Mark S. Popov [1], Nikolay V. Ul'yanovskii [1,2,*] and Dmitry S. Kosyakov [1]

[1] Laboratory of Environmental Analytical Chemistry, Core Facility Center 'Arktika', Northern (Arctic)
Federal University, 163002 Arkhangelsk, Russia; m.popov@narfu.ru (M.S.P.); d.kosyakov@narfu.ru (D.S.K.)
[2] Federal Center for Integrated Arctic Research, 163000 Arkhangelsk, Russia
* Correspondence: n.ulyanovsky@narfu.ru

Contents

Figure S1. Comparison of the analyte recoveries in AWASP at aqueous phase pH 5 and 12 (extractant – dichloromethane, dehydrating agent – Na_2SO_4)

Figure S2. GC-MS chromatogram (SIM detection mode) of the model mixture of target analytes (peak numbers correspond to compound numbers in tables)

Table S1. List of analytes and their physico-chemical properties

Table S2. Accuracy of the developed AWASP-GC-MS method estimated by spike recovery test at 20 mg L^{-1} level with three aqueous matrices – Milli-Q water, river water (Sample 1) and peat bog soil aqueous extract (Sample 2).

Table S3. Accuracy of the developed AWASP-GC-MS method estimated by spike recovery test at 2 mg L^{-1} level with three aqueous matrices – Milli-Q water, river water (Sample 1) and peat bog soil aqueous extract (Sample 2).

Table S4. Accuracy of the developed AWASP-GC-MS method estimated by spike recovery test at 0.2 mg L^{-1} level with three aqueous matrices – Milli-Q water, river water (Sample 1) and peat bog soil aqueous extract (Sample 2).

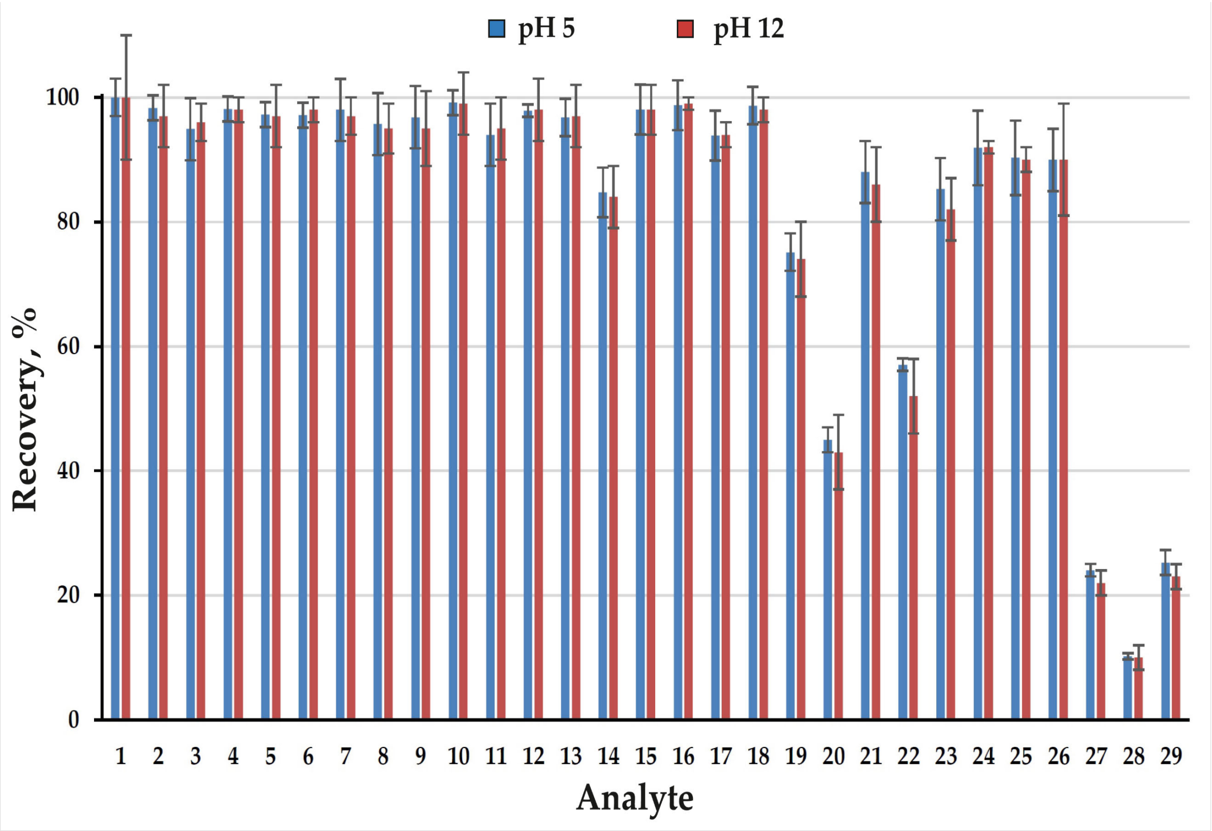

Figure S1. Comparison of the analyte recoveries in AWASP at aqueous phase pH 5 and 12 (extractant – dichloromethane, dehydrating agent – Na_2SO_4)

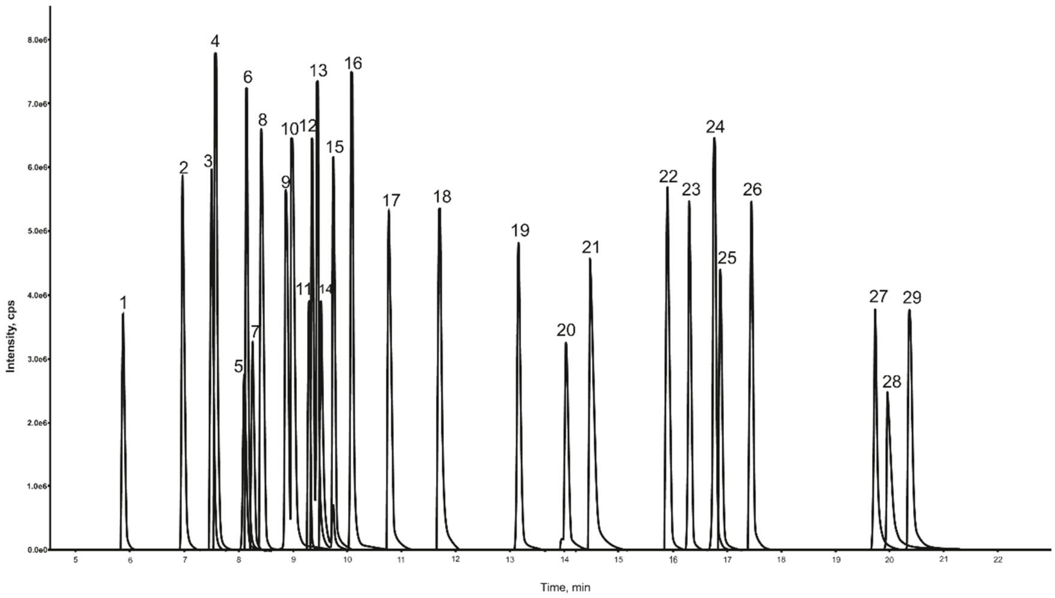

Figure S2. GC-MS chromatogram (SIM detection mode) of the model mixture of analytes (peak numbers correspond to compound numbers in tables)

Table S1. List of analytes and their physico-chemical properties (according to ChemSpider and PubChem databases)

No.	Compound	CAS	Elemental composition	MW, Da	Boiling point, °C	Log*P*	pK_a*
1	1,1,4,4-Tetramethyl-2-tetrazene (TMT)	39247-67-1	$C_4H_{12}N_4$	116	111	0.22	–**
2	Pyridine	110-86-1	C_5H_5N	79	115	0.65	5.23
3	Pyrazine	290-37-9	$C_4H_4N_2$	80	115	-0.26	1.1
4	2-Methylpyridine	109-06-8	C_6H_7N	93	128	1.11	5.96
5	(Dimethylamino)acetonitrile (DMAAN)	926-64-7	$C_4H_8N_2$	84	138	0.1	5.38
6	2,6-Dimethylpyridine	108-48-5	C_7H_9N	107	143	1.65	6.6
7	1-Methyl-1H-pyrazole	930-36-9	$C_4H_6N_2$	82	127	0.167	–
8	2-Methylpyrazine	109-08-0	$C_5H_6N_2$	94	136	0.24	1.42
9	3-Methylpyridine	108-99-6	C_6H_7N	93	144	1.2	5.63
10	4-Methylpyridine	108-89-4	C_6H_7N	93	144	1.19	5.98
11	N-Nitrosodimethylamine (NDMA)	62-75-9	$C_2H_6N_2O$	74	152	-0.57	3.52
12	2,5-Dimethylpyrazine	123-32-0	$C_6H_8N_2$	108	155	0.63	1.59
13	2,6-Dimethylpyrazine	108-50-9	$C_6H_8N_2$	108	154	0.64	–
14	N,N-Dimethylformamide (DMF)	4472-41-7	C_3H_7NO	73	153	-1.01	-0.65
15	2,3-Dimethylpyrazine	5910-89-4	$C_6H_8N_2$	108	156	0.54	1.62
16	2,4,6-Trimethylpyridine	108-75-8	$C_8H_{11}N$	121	170	1.88	7.43
17	3,5-Dimethylpyridine	591-22-0	C_7H_9N	107	184	1.78	6.15
18	2,3,5-Trimethylpyridine	29611-84-5	$C_8H_{11}N$	121	184	2.11	–
19	1-Methyl-1H-1,2,4-triazole	6086-21-1	$C_3H_5N_3$	83	178	-0.86	3.5
20	1-Formyl-2,2-dimethylhydrazine (FADMH)	3298-49-5	$C_3H_8N_2O$	88	97	-0.813	–
21	1-Methyl-1H-imidazole	616-47-7	$C_4H_6N_2$	82	197	-0.209	6.95
22	1H-pyrazole	288-13-1	$C_3H_4N_2$	68	187	0.26	2.48
23	3-Methyl-1H-pyrazole	1453-58-3	$C_4H_6N_2$	82	204	0.777	2.5
24	3,5-Dimethyl-1H-pyrazole	67-51-6	$C_5H_8N_2$	96	218	1.24	1.01
25	4-Methyl-1H-pyrazole	7554-65-6	$C_4H_6N_2$	82	206	0.9	2.91
26	3,4-Dimethyl-1H-pyrazole	2820-37-3	$C_5H_8N_2$	96	218	1.237	–
27	2,4-Dimethyl-1H-imidazole	930-62-1	$C_5H_8N_2$	96	266	0.09	8.48
28	1H-imidazole	288-32-4	$C_3H_4N_2$	68	256	-0.08	6.92
29	4-Methyl-1H-imidazole	822-36-6	$C_4H_6N_2$	82	263	0.3	7.49
IS***	Pyridine-d_5	7291-22-7	C_5D_5N	84	115	0.73	5.23

* - for conjugated acid $(M+H)^+$; ** - no data available; *** - internal standard.

Table S2. Accuracy of the developed AWASP-GC-MS method estimated by spike recovery test at 20 mg L^{-1} level with three aqueous matrices – Milli-Q water, river water (Sample 1) and peat bog soil aqueous extract (Sample 2).

No	Analyte	Spiked, mg L^{-1}	Found, mg L^{-1}			Accuracy, %		
			Milli-Q water	Sample 1	Sample 2	Milli-Q water	Sample 1	Sample 2
1	TMT	20.0	20.7±0.6	20.4±1.2	19.1±1.8	104±3	102±6	96±9
2	Pyridine	23.0	23.3±0.2	22.9±1.2	21.3±1.5	101±1	100±5	93±7
3	Pyrazine	23.2	23.6±1.3	23.2±2.0	23.9±0.9	102±5	100±9	103±4
4	2-Methylpyridine	28.4	29.6±1.3	27.5±1.1	27.0±1.4	104±5	97±4	95±5
5	DMAAN	21.9	22.2±0.3	21.2±1.1	22.1±0.4	101±1	97±5	101±2
6	2,6-Dimethylpyridine	22.6	23.2±0.8	21.3±1.5	22.0±1.4	103±4	94±6	97±6
7	1-Methyl-1H-pyrazole	23.2	24.5±1.1	22.8±2.4	24.6±2.2	105±5	98±10	106±10
8	2-Methylpyrazine	23.7	24.8±1.2	23.1±2.4	25.1±1.5	105±5	98±10	106±6
9	3-Methylpyridine	23.1	23.9±1.3	21.4±2.6	21.7±1.6	103±6	93±11	94±7
10	4-Methylpyridine	23.9	24.8±1.2	21.9±2.7	21.8±2.4	104±5	92±11	92±10
11	NDMA	20.0	20.4±0.9	20.0±0.5	20.4±0.4	102±5	100±3	102±2
12	2,5-Dimethylpyrazine	23.1	24.3±1.8	21.7±2.6	22.9±0.7	105±8	94±11	99±3
13	2,6-Dimethylpyrazine	21.3	22.4±1.0	20.6±2.2	20.6±1.3	105±5	97±10	97±6
14	DMF	22.8	22.9±1.0	21.8±1.4	22.8±1.0	100±5	96±6	100±5
15	2,3-Dimethylpyrazine	24.1	24.6±1.2	23.1±1.3	24.4±1.1	102±5	96±5	101±5
16	2,4,6-Trimethylpyridine	21.9	21.8±1.1	21.0±1.2	21.6±6.6	100±5	96±5	99±3
17	3,5-Dimethylpyridine	22.9	22.7±1.0	22.6±1.1	20.4±0.2	99±4	98±5	89±1
18	2,3,5-Trimethylpyridine	21.5	21.8±1.2	21.4±1.2	20.6±1.5	102±5	100±5	96±6
19	1-Methyl-1H-1,2,4-triazole	25.1	24.9±1.0	24.2±1.1	24.4±1.5	99±4	96±4	97±6
20	FADMH	23.4	22.1±1.1	21.7±2.3	21.7±2.2	95±5	93±10	93±9
21	1-Methyl-1H-imidazole	25.4	25.8±1.1	24.7±1.4	24.6±1.7	102±5	97±5	97±7
22	1H-pyrazole	23.0	23.6±0.8	22.4±1.3	23.4±2.3	103±4	97±6	102±10
23	3-Methyl-1H-pyrazole	23.8	23.3±0.9	22.7±1.7	22.6±1.6	98±4	95±7	95±7
24	3,5-Dimethyl-1H-pyrazole	23.4	23.3±0.9	23.6±1.7	22.8±0.4	100±4	101±7	98±2
25	2,4-Dimethyl-1H-imidazole	23.2	24.2±1.7	22.5±2.0	21.7±1.7	104±7	97±9	94±7
26	4-Methyl-1H-pyrazole	21.4	22.2±1.7	21.4±1.7	20.4±1.4	104±8	100±8	95±6
27	3,4-Dimethyl-1H-pyrazole	22.4	21.7±0.4	22.7±2.1	21.0±1.8	97±2	101±9	94±8
28	1H-imidazole	23.3	22.8±2.2	22±1.3	22.0±1.8	98±9	94±6	94±8
29	4-Methyl-1H-imidazole	22.3	20.5±0.6	22.0±2.3	21.1±2.2	92±3	99±10	95±10

Table S3. Accuracy of the developed AWASP-GC-MS method estimated by spike recovery test at 2 mg L^{-1} level with three aqueous matrices – Milli-Q water, river water (Sample 1) and peat bog soil aqueous extract (Sample 2).

No	Analyte	Spiked, mg L^{-1}	Found, mg L^{-1}			Accuracy, %		
			Milli-Q water	Sample 1	Sample 2	Milli-Q water	Sample 1	Sample 2
1	TMT	2.00	1.95±0.10	1.91±0.15	1.85±0.15	98±5	99±5	93±8
2	Pyridine	2.30	2.36±0.18	2.30±0.11	2.19±0.17	103±8	100±8	95±7
3	Pyrazine	2.32	2.37±0.04	2.23±0.10	2.15±0.16	102±2	96±4	93±7
4	2-Methylpyridine	2.84	2.82±0.05	2.80±0.06	2.70±0.12	99±2	99±2	95±4
5	DMAAN	2.19	2.07±0.20	2.16±0.14	2.02±0.13	95±9	99±6	92±6
6	2,6-Dimethylpyridine	2.26	2.25±0.10	2.28±0.15	2.20±0.10	100±4	101±9	97±4
7	1-Methyl-1H-pyrazole	2.32	2.34±0.13	2.31±0.15	2.21±0.18	101±6	100±6	95±8
8	2-Methylpyrazine	2.37	2.29±0.15	2.30±0.12	2.19±0.17	97±6	97±5	92±7
9	3-Methylpyridine	2.31	2.38±0.14	2.26±0.08	2.25±0.12	103±6	98±6	97±5
10	4-Methylpyridine	2.39	2.24±0.22	2.36±0.24	2.16±0.15	94±9	99±10	90±10
11	NDMA	2.00	1.92±0.08	1.94±0.12	1.90±0.18	96±4	97±6	95±9
12	2,5-Dimethylpyrazine	2.31	2.32±0.07	2.27±0.10	2.22±0.10	100±3	98±4	96±4
13	2,6-Dimethylpyrazine	2.13	1.98±0.15	2.08±0.14	2.00±0.14	93±7	98±6	94±7
14	DMF	2.28	2.19±0.10	2.19±0.12	2.16±0.12	96±5	96±5	95±5
15	2,3-Dimethylpyrazine	2.41	2.46±0.08	2.40±0.15	2.21±0.18	102±3	100±6	92±8
16	2,4,6-Trimethylpyridine	2.19	2.07±0.13	2.06±0.13	2.00±0.16	95±6	94±6	91±7
17	3,5-Dimethylpyridine	2.29	2.19±0.14	2.15±0.15	2.09±0.15	96±6	94±6	91±7
18	2,3,5-Trimethylpyridine	2.15	2.07±0.10	2.14±0.10	2.04±0.11	96±5	99±5	95±5
19	1-Methyl-1H-1,2,4-triazole	2.51	2.62±0.15	2.41±0.08	2.35±0.15	104±6	96±3	94±6
20	FADMH	2.34	2.18±0.20	2.15±0.21	2.07±0.19	93±9	92±9	88±8
21	1-Methyl-1H-imidazole	2.54	2.43±0.14	2.38±0.17	2.34±0.12	96±6	94±7	92±5
22	1H-pyrazole	2.30	2.19±0.20	2.20±0.19	2.15±0.16	95±9	96±8	93±7
23	3-Methyl-1H-pyrazole	2.38	2.51±0.15	2.26±0.13	2.14±0.18	105±6	95±5	90±8
24	3,5-Dimethyl-1H-pyrazole	2.00	1.90±0.10	1.91±0.13	1.89±0.12	95±5	96±5	95±6
25	2,4-Dimethyl-1H-imidazole	2.30	2.36±0.18	2.26±0.20	2.10±0.20	103±8	95±9	91±9
26	4-Methyl-1H-pyrazole	2.32	2.37±0.07	2.36±0.11	2.19±0.12	102±3	102±5	94±5
27	3,4-Dimethyl-1H-pyrazole	2.84	2.82±0.13	2.62±0.25	2.58±0.30	99±5	92±9	91±11
28	1H-imidazole	2.19	2.02±0.20	1.92±0.28	1.87±0.25	92±9	88±13	85±11
29	4-Methyl-1H-imidazole	2.26	2.05±0.23	2.00±0.30	1.95±0.27	91±10	88±10	86±13

Table S4. Accuracy of the developed AWASP-GC-MS method estimated by spike recovery test at 0.2 mg L^{-1} level with three aqueous matrices – Milli-Q water, river water (Sample 1) and peat bog soil aqueous extract (Sample 2).

No	Analyte	Spiked, mg L^{-1}	Found, mg L^{-1}			Accuracy, %		
			Milli-Q water	Sample 1	Sample 2	Milli-Q water	Sample 1	Sample 2
1	TMT	0.201	0.197±0.009	0.185±0.015	0.163±0.024	98±5	92±8	81±12
2	Pyridine	0.230	0.219±0.012	0.229±0.013	0.210±0.019	95±5	100±6	91±8
3	Pyrazine	0.230	0.230±0.010	0.210±0.018	0.172±0.019	100±4	91±8	75±8
4	2-Methylpyridine	0.284	0.280±0.014	0.271±0.012	0.235±0.025	99±2	95±4	83±9
5	DMAAN	0.222	0.228±0.018	0.198±0.015	0.184±0.023	103±8	89±7	83±10
6	2,6-Dimethylpyridine	0.231	0.238±0.020	0.211±0.020	0.194±0.011	103±9	91±9	84±5
7	1-Methyl-1H-pyrazole	0.232	0.235±0.009	0.222±0.011	0.179±0.015	101±4	96±5	77±6
8	2-Methylpyrazine	0.238	0.231±0.010	0.224±0.012	0.197±0.014	97±4	94±5	83±6
9	3-Methylpyridine	0.227	0.221±0.013	0.210±0.018	0.175±0.013	97±6	93±8	77±6
10	4-Methylpyridine	0.241	0.247±0.017	0.226±0.012	0.206±0.015	102±7	94±5	85±6
11	NDMA	0.201	0.205±0.010	0.185±0.015	0.165±0.019	102±5	92±8	82±9
12	2,5-Dimethylpyrazine	0.226	0.219±0.014	0.212±0.010	0.172±0.021	97±6	94±4	76±10
13	2,6-Dimethylpyrazine	0.210	0.209±0.012	0.191±0.017	0.175±0.015	100±6	91±8	83±7
14	DMF	0.229	0.219±0.019	0.210±0.018	0.184±0.024	96±5	92±8	80±5
15	2,3-Dimethylpyrazine	0.241	0.240±0.017	0.221±0.017	0.188±0.025	100±7	92±7	78±10
16	2,4,6-Trimethylpyridine	0.219	0.215±0.014	0.207±0.014	0.181±0.012	98±7	95±6	83±5
17	3,5-Dimethylpyridine	0.231	0.225±0.015	0.219±0.012	0.189±0.018	97±6	95±5	82±8
18	2,3,5-Trimethylpyridine	0.210	0.216±0.010	0.193±0.018	0.158±0.020	103±5	92±9	75±10
19	1-Methyl-1H-1,2,4-triazole	0.248	0.245±0.025	0.224±0.015	0.192±0.019	99±10	90±6	77±8
20	FADMH	0.232	0.220±0.021	0.200±0.025	0.168±0.033	95±9	86±11	72±14
21	1-Methyl-1H-imidazole	0.252	0.250±0.020	0.240±0.014	0.179±0.021	100±8	95±6	71±8
22	1H-pyrazole	0.227	0.217±0.014	0.199±0.018	0.178±0.022	96±6	88±8	78±10
23	3-Methyl-1H-pyrazole	0.239	0.228±0.016	0.220±0.014	0.189±0.024	95±7	92±5	79±10
24	3,5-Dimethyl-1H-pyrazole	0.231	0.227±0.011	0.218±0.012	0.188±0.017	98±5	96±5	81±7
25	2,4-Dimethyl-1H-imidazole	0.230	0.216±0.015	0.219±0.013	0.181±0.020	94±7	95±6	79±9
26	4-Methyl-1H-pyrazole	0.214	0.226±0.018	0.208±0.018	0.177±0.025	106±8	97±8	83±12
27	3,4-Dimethyl-1H-pyrazole	0.225	0.240±0.015	0.201±0.034	0.152±0.034	106±7	89±13	67±15
28	1H-imidazole	0.232	0.220±0.026	0.210±0.025	0.134±0.028	95±11	91±11	58±12
29	4-Methyl-1H-imidazole	0.224	0.205±0.029	0.187±0.032	0.157±0.041	92±13	83±14	70±18
